# The effect of pond dyes on oviposition and survival in wild UK *Culex* mosquitoes

**DOI:** 10.1371/journal.pone.0193847

**Published:** 2018-03-28

**Authors:** Natali Ortiz-Perea, Rebecca Gander, Oliver Abbey, Amanda Callaghan

**Affiliations:** Ecology and Evolutionary Biology Section, School of Biological Sciences, University of Reading, Whiteknights, Reading, United Kingdom; Fundacao Oswaldo Cruz, BRAZIL

## Abstract

British *Culex pipiens* complex [*Culex pipiens* sensu lato) mosquito distribution, abundance, and potential for disease transmission are intimately linked to their environment. Pond and lake dyes that block light to restrict algal photosynthesis are a relatively new product assumed to be an environmentally friendly since they are based on food dyes. Their use in urban garden ponds raises questions linked to mosquito oviposition, since coloured water can be an attractant. *Culex (*mostly *pipiens)* is commonly found in UK gardens and is a potential vector of viruses including the West Nile Virus (WNV). Any factors that significantly change the distribution and population of *Cx pipiens* could impact future risks of disease transmission. A gravid trap was used to catch female *Cx pipiens* mosquitoes for use in oviposition choice tests in laboratory and semi-field conditions. Two types of pond dye, blue and shadow (which looks slightly red), were tested for their impact on oviposition and survival of wild caught *Cx pipiens*. There were no significant differences in the number of egg batches laid when gravid mosquitoes were given a choice between either blue dye and clear water or shadow dye and clear water indicating that these dyes are not attractants. Larvae hatched from egg batches laid by wild-caught gravid females were used to measure survival to adulthood with or without dye, in a habitat controlled to prevent further colonisation. The experiment was run twice, once in the summer and again in the autumn, whereas the dyes had no impact on emergence in the summer, there were highly significant reductions in emergence of adults in both dye treated habitats in the autumn. Containers with or without shadow dye were placed outside to colonise naturally and were sampled weekly for larvae and pupae over a 6 month period through summer and autumn. There was a significant negative effect of shadow dye on pupal abundance in a three week period over the summer, but otherwise there was no effect. It is likely that population abundance and food was a more powerful factor for mosquito survival than the dye.

## Introduction

The last 15 years have seen an unprecedented change in the status of vector-borne disease in Europe as a result of multiple and complex environmental changes influencing mosquito populations [1, 2]. There are many examples throughout history, in temperate and tropical countries worldwide, of how changes to human activities, e.g. deforestation, agricultural practices and urbanisation, alter the distribution, ecology or behaviour of a disease vector and create the environmental conditions conducive to disease transmission [2]. In the UK, changes to land use, climate change and human activities in adaption to that change, are likely to affect mosquito populations. This provides a compelling rationale to investigate how we impact mosquito ecology and behaviour, especially considering their potential as vectors of diseases. It is against this backdrop that we have been studying urban artificial containers (such as water butts) and small ponds which are ideal habitats for a number of mosquito species [3].

In previous studies, we investigated mosquito populations in water butts in both urban and rural habitats [3, 4] where we found a marked difference in mosquito species composition and abundance, with *Culex pipiens* (*pipiens*) dominating urban habitats [3]. We concluded that the storage of water in domestic gardens was increasing urban populations of *Culex pipiens*, a potential vector of West Nile Virus (WNV). Water butts are not the only artificial water bodies in gardens; many gardens have ponds. In the UK domestic gardens are estimated to contain 2.5–3.5 million ponds [5], forming important reservoirs for taxa and helping to sustain aquatic biodiversity [6]. Where ponds are developed as wildlife refugia, the lack of voracious fish predators means that mosquitoes can reach high densities. Understanding factors that will impact on mosquito numbers is important information in the bank for any future issues with mosquito control.

Despite their potential importance for wildlife, domestic ponds are often managed for aesthetic purposes and difficulties arise in maintaining normal ecosystem function whilst retaining desirable aesthetic qualities [7]. An example is the current fashion for using pond dyes to improve reflection and reduce algal growth. Pond and lake dyes are a relatively new commercially available product, sold as an environmentally friendly way to stop the growth of algae through the disruption of photosynthesis [8]. They have proved to be popular at recent high profile garden shows such as Chelsea and Hampton Court. One such product on the market is produced by DyoFix who state that their pond dyes are a blend of European food approved colour dye. The mode of action explained by the manufacturer is that it acts as a light filter, stopping colours on the red end of the spectrum from penetrating the water. Since the plant pigment chlorophyll *a*, which is crucial to photosynthesis, absorbs red light at 662nm, the theory is that addition of a red dye filter will prevent red light from reaching algae below the surface of the water, thereby inhibiting photosynthesis.

The concept of using dyes to limit algal growth by surface inhibition has been around for many years with an early example being aniline dye [9]. Whilst effective at reducing blue-green algal growth it was a particularly hazardous chemical and was never intended for practical use. Modern pond dyes, however, claim to be environmentally friendly with manufacturers stating that they can be used not only at a domestic level in residential ponds, but also have commercial application, being able to work on large bodies of water such as lakes. The manufacturers of pond dyes are confident that they are environmentally friendly since they meet European Food Additive regulations, although very little actual toxicity information is available [8]. A few studies exist that look at the impact of wavelength- blocking pond dyes on algal growth, with mixed results. One found no significant impact on phytoplankton growth, with no difference in chlorophyll a concentrations at the concentrations of dye used (Aquasure, 4ml/m3) [10] whilst another found no reduction in microalgae growth until dye was applied at a high concentration (Aquashade, 5ml/m3 [11]

However there are even fewer studies investigating the use of the product on freshwater fauna, and no investigations into any secondary non-lethal effects the dye may have on organisms, with the exception of a PhD thesis [12] where a dye (Crystal Blue-Ocean) had no impact on catfish survival and yield. Incidentally this study also failed to find any difference in algal growth between dye treated and untreated water.

The use of pond dyes in domestic and ornamental gardens raises questions linked to mosquito oviposition, since coloured water can be an attractant [4]. *Culex pipiens* is commonly found in UK gardens and is a potential vector of viruses including the West Nile Virus [13, 14]. Whilst currently there is no evidence of disease transmission in the UK, any factors that significantly change the distribution and population of *Cx pipiens* could impact future risks of disease transmission. Our previous work demonstrated that *Cx*. *pipiens* females prefer to lay eggs in black dye water compared with the control in the laboratory and semi-field conditions. It was also observed that survival of larvae through to adults was significantly reduced in dyed water, suggesting that there is some form of chronic toxicity [4]. These results suggest that the dyes are in fact not as environmentally friendly as previously suggested. It also raises the possibility that pond dyes could attract mosquitoes to lay eggs in garden ponds. Studies have reported that *Culex* sp. females use water reflection, darkness, temperature, pheromones and kairomones as part of the cues to choose an oviposition site [15]. There is also evidence that mosquito oviposition is influenced by water body or container colour, type and size [16–19].

Blue pond and shadow lake dyes (red colour) are products similar to the black pond dye which blocks the red end of the visible light spectrum penetrating the water. These dyes were created to be more natural than the black colour pond when applied to the water and are less reflective [20]. Pond blue is the most popular dye used and the most economic although lake shadow is a popular product because it is a colourless dye in the water [20].

Our previous work demonstrated that Dyofix black dye was an attractant to gravid mosquitoes with a significant impacts on survival (emergence) but no measureable impact on mosquito numbers in a semi-natural habitat [4]. The lack of an impact in a natural habitat was explained by a balance between higher oviposition but reduced survival in a black dye treated habitat. Based on this hypothesis, two further pond dyes were studied to determine whether the impact was one found generally or whether the impact of pond dyes on mosquito numbers varied depending on the dye.

## Methods

### Trapping wild gravid females

**Fig 1:**
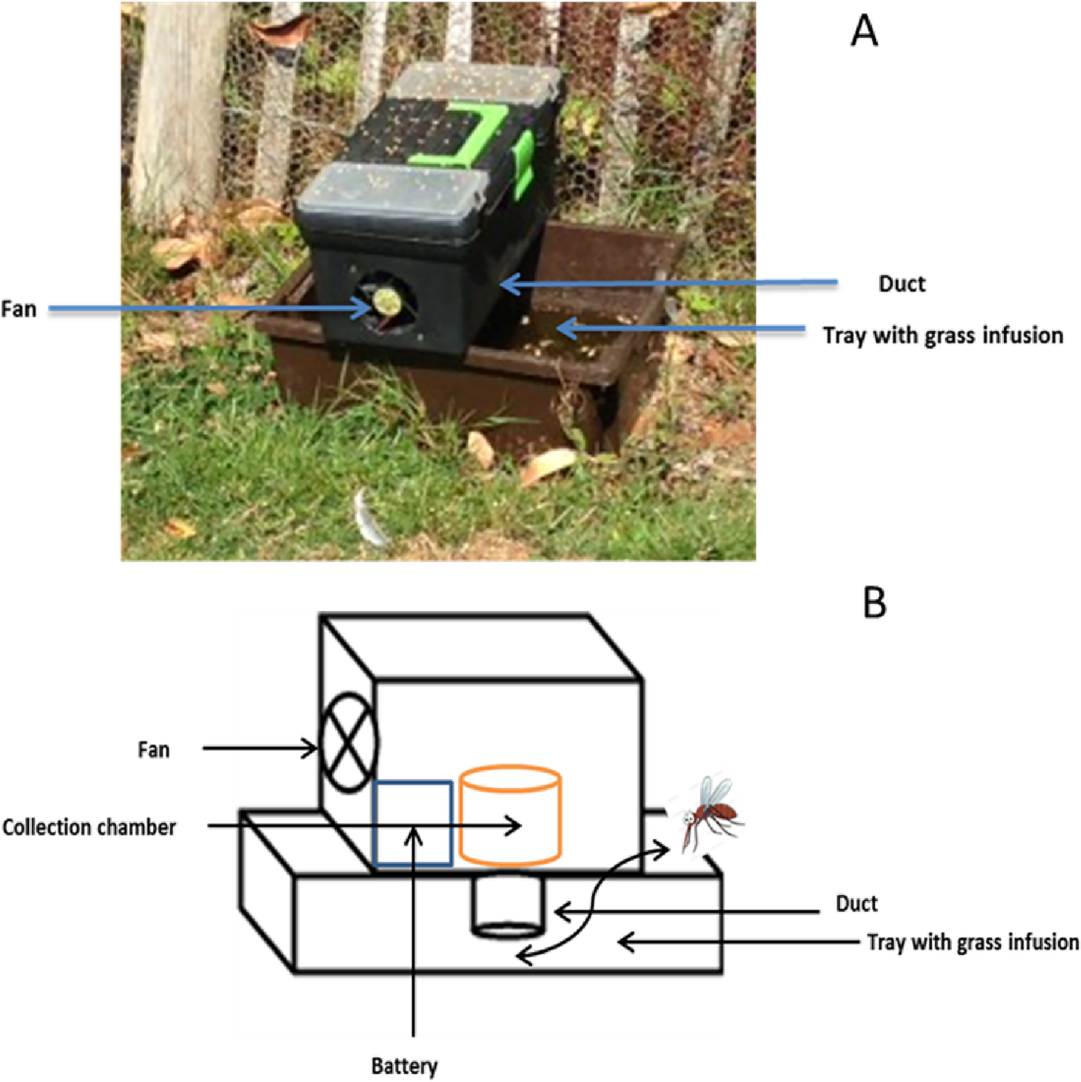
A modified emergence trap [3] based on Reiter’s gravid box trap design. **A**. A diagram of the ovitrap. **B**. Ovitrap used during the experiments.

Wild gravid female *Culex* were collected using Reiter ovitraps [21] modified by [3] (Fig 1A). Attracted to the bait infusion, females are pulled into a duct connected to the collection chamber by use of a fan located in the upper portion of the trap. The fan is connected to a valve-regulated lead-acid battery that produces a negative air pressure inside the box, allowing for mosquito collection (Fig 1B). The ovitrap consisted of two parts; a lightweight upper portion (a modified toolbox containing a fan, battery and trap for the adults) and a lower portion (5 litre tray) which contained the attractant infusion. Infusions were prepared by fermenting 1 lb of freshly cut grass, 1 lb of hay, 5 g of brewer’s yeast and 60 L of tap water. The mixture was fermented in an 80 L black waste bin (44.5cm x 58.5 cm) outdoors for 7 days at the University of Reading. Prior to use the infusion was filtrated using a metallic ring that at the bottom presents a net to remove the grass and the hay.

In total, 10 traps were used for this study, placed in the glasshouse area of the Whiteknights campus of the University of Reading, Berkshire, England (51.4419° N, 0.9456° W). Gravid female mosquitoes were collected in summer (July to August) in 2014 and 2015. Approximately 1000 gravid female mosquitoes were collected through the sampling period in 2014 and 2015. Most of the mosquitoes sampled belong to the *Culex* genus. However, a few number of *Anopheles plumbeus* (<5) and *Culiseta annulata* (<5) were also present throughout the trapping period.

### Pond dyes

Two pond dyes (SGP Blue and SGP Shadow (Dyofix)) were used in this study, supplied as odourless solutions. Pond Blue has a pH of 5–6 at 10g/L water and a rat oral LD50 of 2g/Kg and fish LD50 of >100mg/L, and Lake Shadow has a pH of 7–8 at 10g/L water, a rat oral LD50 of 2g/Kg and fish LC50 of >100mg/L [22].

### Oviposition preferences of wild mosquitoes

A choice experiment was performed between July to September 2014 and again in 2015. A tent (245 x 145 x 95 cm) was placed adjacent to a wooded area in the same location as the gravid traps. Approximately, two hundred gravid mosquitoes were collected from the modified traps and transferred into the tent 24 h post collection. Adult females were provided with a 10% sucrose solution. The tent contained 14 2 L plastic containers (14 length x 21width x 10 height cm): 7 with 1.5 L tap water and 7 with 1.5 L water treated with either blue or shadow pond dye [8]. A randomised block design was used to remove edge effects. After seven days, the containers were taken to the laboratory to count egg batches laid in each container. The experiment was repeated two times and with two types of dye: blue and shadow. Treatments were tap water, 1.5 μl of blue or 1.5 μl of shadow Dyofix pond dye/ 1.5L.

The choice experiment was repeated with wild-caught gravid females in laboratory conditions (25 ± 2°C) and normal light/dark photoperiod (16:8 h). Twenty gravid females were chosen randomly and transferred into each of 5 cotton net cages 30 x 30 x 30 cm per treatment set. Each cage contained a 10% sucrose solution and two 200 ml transparent plastic cups (12 x 5 cm) filled with 150 ml of either tap water or dye water (10 μ1 blue or 10 μl shadow). The plastic cups in each cage were rotated 90° daily to eliminate positional effect and collection of eggs was begun the day after the experiment set up. Eggs were removed from the plastic cups in each cage daily to eliminate oviposition effect. Treatments were tap water and blue and shadow Dyofix pond dye. 10 μl of blue or 10 μl of shadow were dissolved in 1L of tap water and then transferred to the 5 200 ml plastic cups.

The choice experiments were repeated as above but in the absence of light; black bags were used as a cover in each cage during the experiment.

### Emergence study

**Fig 2:**
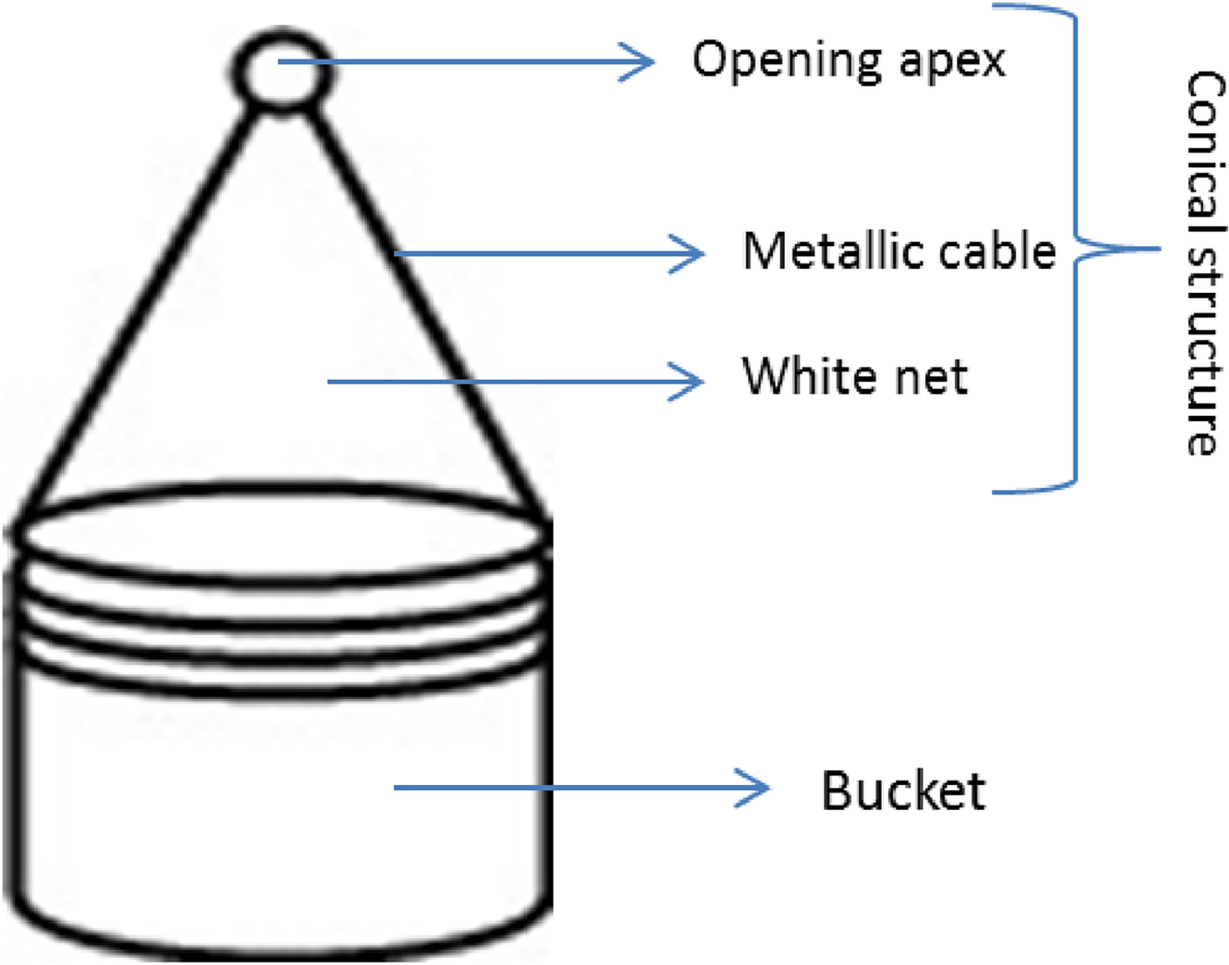
A modified emergence trap.

A modified emergence trap [23] was used to measure the impact dye had on survival (Fig 2). Traps were made from lidded 11 litre cylindrical plastic bins (23 x 28 cm). The surface of each lid was removed, keeping the peripheral edges (ring) connected to the bin. Four holes were punched on each ring where two metallics cables were glued to create a conical structure. The conical structure was covered with a white net with an opening in the apex to remove adults. Each bin (9 replicates per treatment) was filled with 10 litres of tap water; a hundred wild larvae (above 2^nd^ instar) and 1.2 g of guinea pig food (3mm pellets). The wild larvae were obtained from egg batches collected from wild mosquito females. Treatments were tap water, 10 μl of blue or 10 μl of shadow of liquid Dyofix pond dye in 10L water [22]. The bins were placed in 9 sites at the glasshouse area (51°26’13.2”N; 0°56’31.2”W).

Traps were monitored daily for adult mosquitoes. These were captured using a manual aspirator and transferred into small plastic tubes. The emergence experiment was repeated twice. The first experiment was performed on 27^th^ August—8 ^th^ September (Summer) and the second experiment was set up on 29^th^ September—16 ^th^ November 2015 (Autumn).

### Natural colonisation of containers with or without dyes

Eighteen 10 L bins (26 cm x 26 cm) were filled with 8 L of tap water or 8 L of tap water and shadow dye and 4 g of oak leaves tree collected from the Harris Garden at the University of Reading. For the dye treatment, 8 μl of dye was added to each container (i.e. 1μl concentrate/L water). The bins were placed in pairs in the experimental grounds of the School of Agriculture at the University of Reading (51.4419°N, 0.9456°W).

Containers were sampled weekly from August 11^th^ 2014 until 12^th^ November 2014. Sampling was carried out using an aquarium fish net (6 x 12 cm; 1 mm mesh). The net was dropped into each container and moved in circles from the top to the bottom for 10 seconds before removal. The larvae and pupae collected were transferred to a tray where they were counted; the larvae were returned to the container. Pupae sampled with the net were placed in plastic tubes using plastic pipettes and taken to the laboratory to complete their development to adults. In addition to the net sampling, a visual search for pupae was performed to remove all pupae in each container.

### Statistical analysis

Data were tested for normality using a Shapiro-Wilk normality test. Where data were normally distributed a paired t-test and one-way analysis of variance (ANOVA) was used. To observe the difference between the number of egg batches laid by gravid females and the preference in colour a paired t-test was performed. One-way analysis of variance (ANOVA) was used to compare egg batches laid in darkness and light. Oviposition in the tent was not normally distributed and a nonparametric Mann–Whitney *U*-test was performed to assess differences in the number of egg batches laid by blood feed mosquito in semi-field conditions.

Differences in adult emergence between treatments were analysed using a generalised linear model binomial test. Abundance data in water butts were Log (x+1) transformed and the relationship between mosquito abundance and treatment analysed using a 2 way repeated measures ANOVA (Analysis of variance). Mosquito abundance among untreated and treated bins was analysed through the period of time and across the season (summer and autumn). We performed all statistical analysis using R version 3.3.1 [24].

## Results

### Oviposition selection in a tent

**Fig 3:**
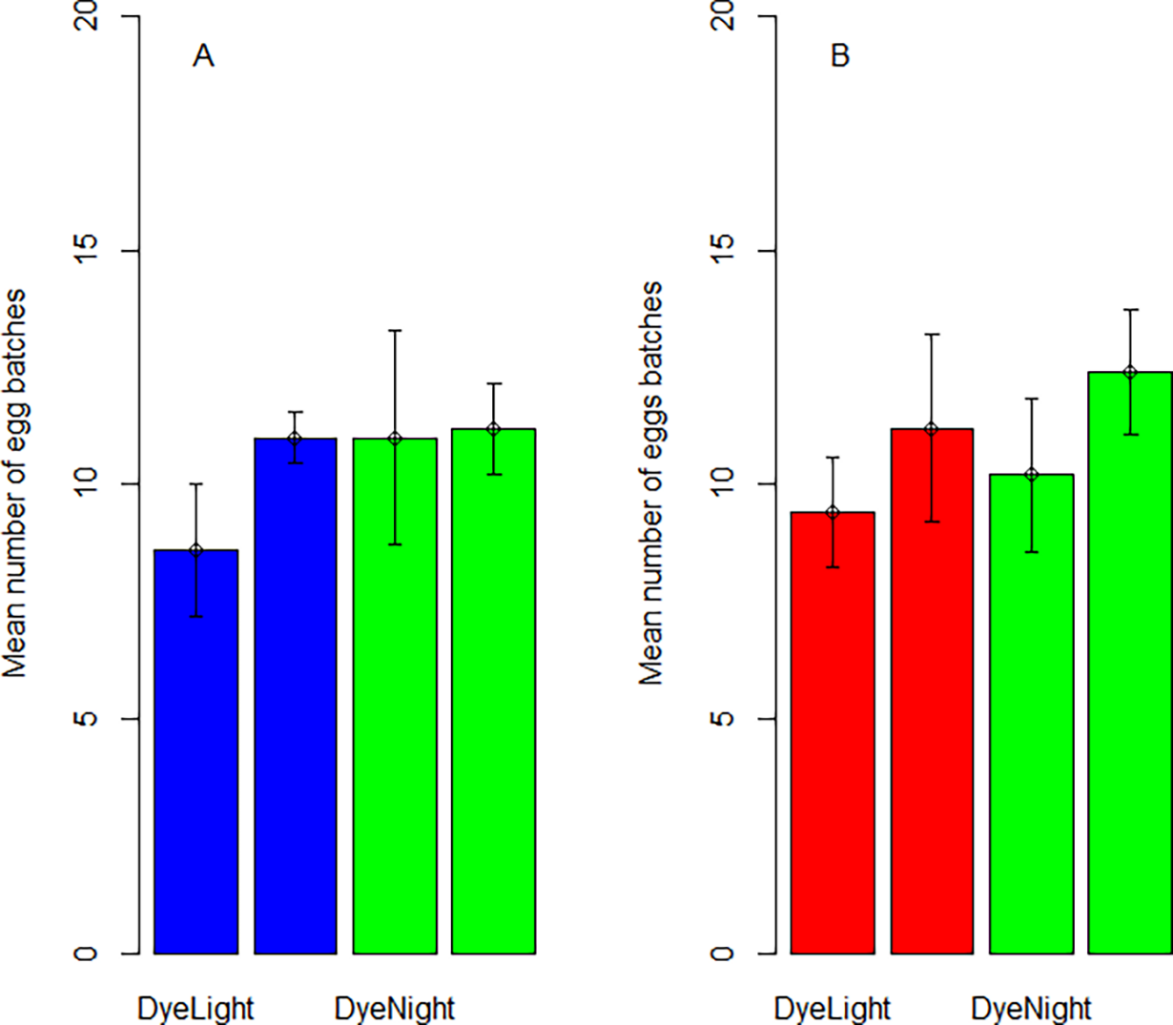
Mean number of egg batches (±SE) laid by *Culex* spp. in semi-field conditions. **A.** Differences between blue colour dye and tap water. **B.** Differences between shadow colour dye and tap water.

The total number of eggs batches laid by the ovipositing female mosquitoes were 593; 386 in (65.1%) treated treatments and 207 in (34.9%) in untreated treatment (S1 Table). Despite the difference in numbers, statistical analysis found no preference for either the blue or shadow dye compared to the tap water (Shadow W = 126.5; *P* = 0.185 (Fig 3A); Blue W = 135; P = 0.093 (Fig 3B).

### Oviposition behaviour in laboratory

**Fig 4:**
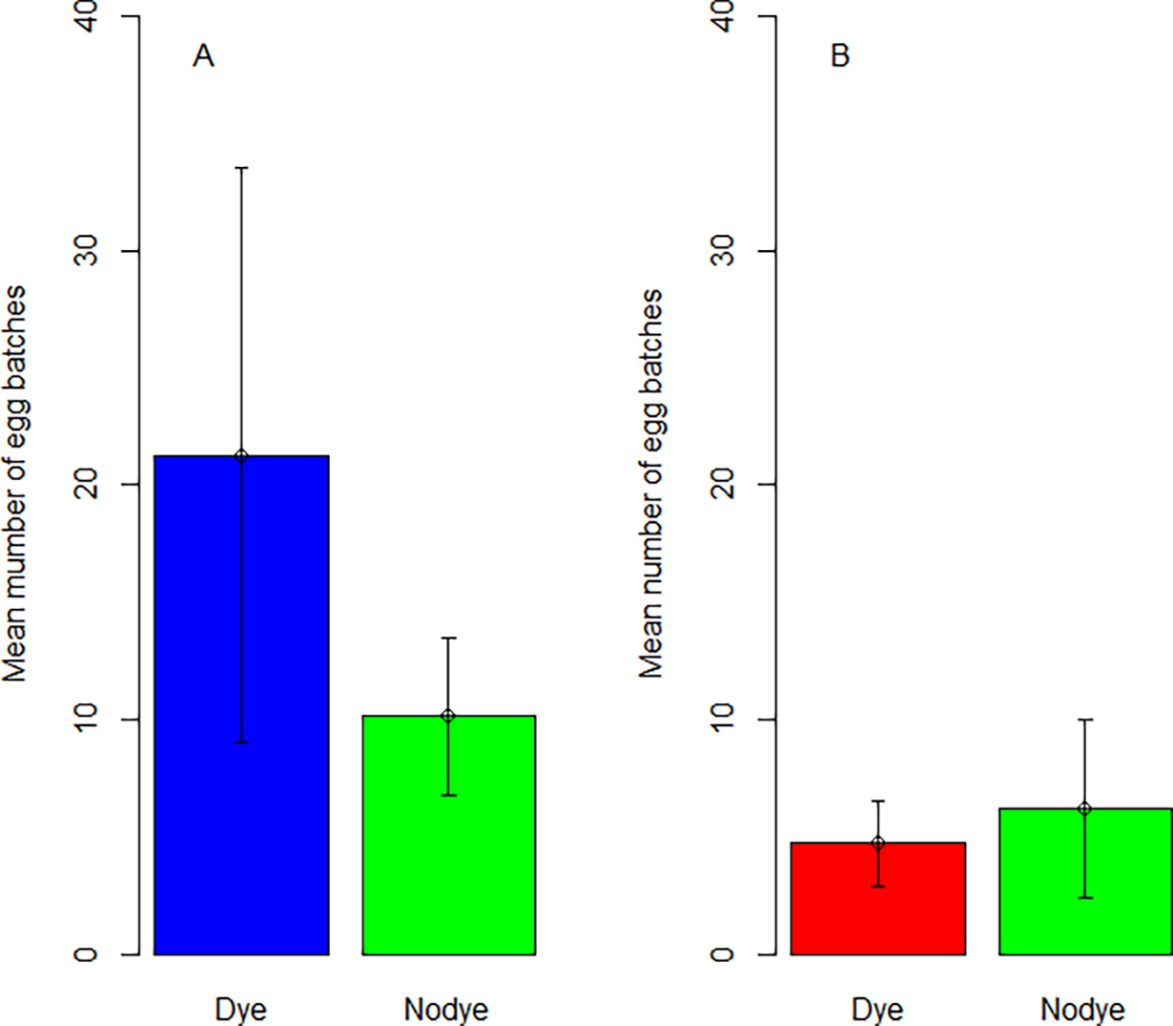
Mean number of eggs batches (±SE) laid by *Culex* wild mosquito in temperature control room in normal and darkened conditions. **A.** blue colour dye and tap water. **B**. shadow colour dye and tap water.

*Culex* spp. showed no preference for oviposition in blue or shadow colour dye in normal light/dark (blue t = -1.776; df = 8; *P* = 0.114; shadow t = -0.919; df = 8; *P* = 0.385) or in darkened conditions (blue t = 0.219; df = 8; *P* = 0.832 (Fig 4A); shadow t = -0.888; df = 8; *P* = 0.400 (Fig 4B, S1 Table)).

### Emergence study: field conditions

**Fig 5:**
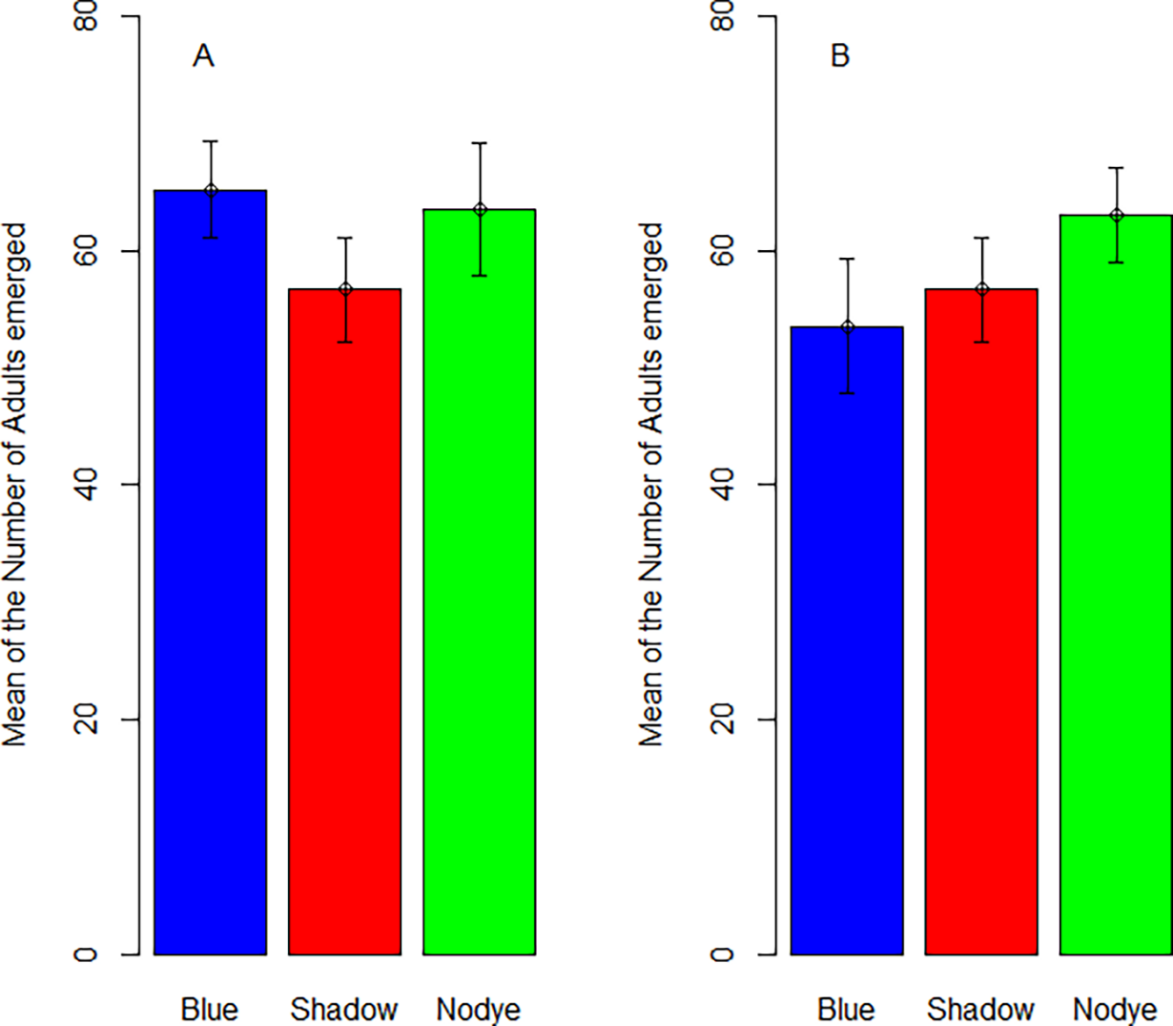
Mean number (±SE) of adults emerged from the three treatments (tap, blue and shadow) in A. summer and B. autumn.

The total number of adults emerging from different treatments was not significant in the summer (Z = -1.259, *P* = 0.208) but varied significantly in the autumn (Z = -4.049, *P* < 0.001) (Fig 5). A Tukey post-hoc analysis found significant differences between the shadow dye and untreated bins and between the blue dye and untreated bins in the autumn season (Table 1, S2 Table).

**Table 1:** Tukey post doc tests comparing adult emergence from the three treatments (tap, blue and shadow) in summer and autumn.

Interaction	Summer		Autumn	
	Z	*P*	Z	*P*
Blue-Shadow	0.738	0.741	-1.327	0.38
Blue-Tap	-1.262	0.417	-4.056	<0.001
Shadow-Tap	-2.001	0.112	-2.738	0.017

### Natural colonisation of containers

**Fig 6:**
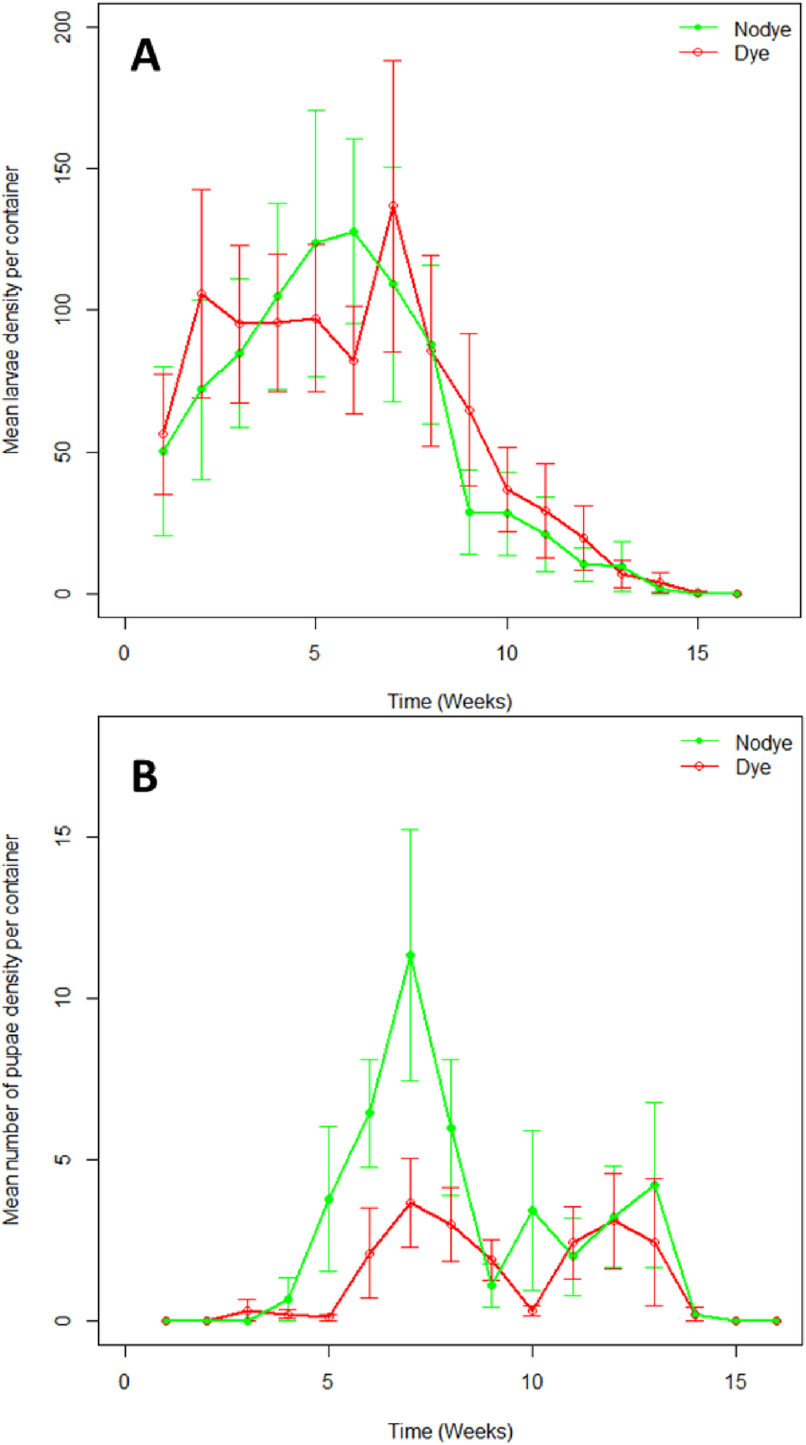
Mean (±SEM) *Culex pipiens* abundance in untreated and shadow dye-treated small bins across the sampling period. **A**. larvae. **B.** pupae.

**Fig 7:**
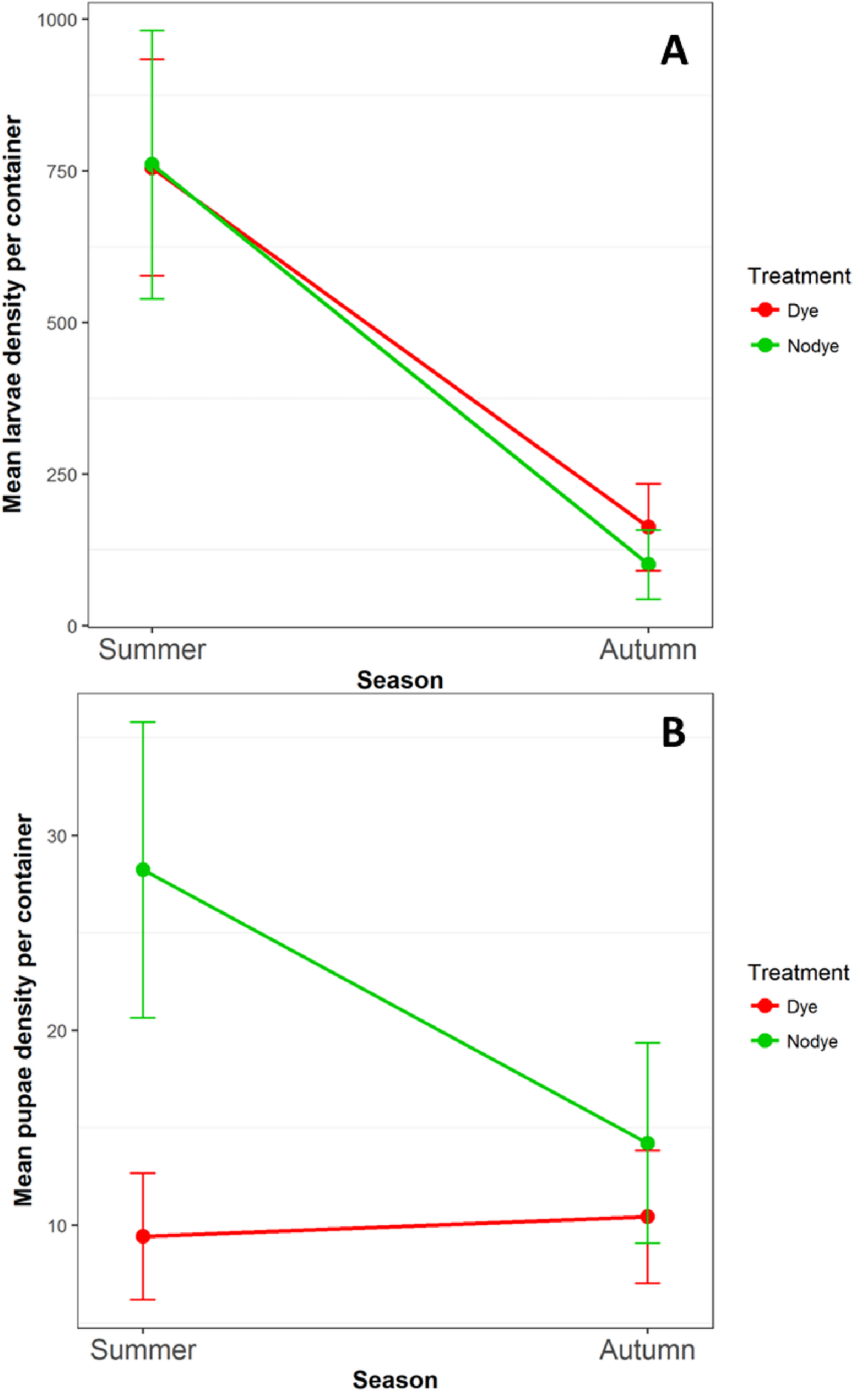
Mean (±SEM) abundance between untreated and treated small bins. **A**. larvae. **B**. Pupae.

The only species recorded breeding in the small experimental bins were *Culex pipiens (sensu lato)*. Larval and pupal numbers were analysed by season; summer (August-Middle of September) and autumn (Middle to September to October). No significant differences were observed in overall larval and pupal densities between treatments (larval F _1 32_ = 0.318, *P* = 0.576; pupal F _1 33_ = 2.611 *P* = 0.116) (Fig 6). However untreated bins showed overall higher pupal densities in summer season compared with treated bins (F _1 16_ = 6.317, *P* = 0.023) (Fig 7A). This was because pupal density varied significantly in weeks 5, 6 and 7 between treated and untreated bins (F _1 16_ = 5.254, *P* = 0.036). The total number of larvae varied significantly between seasons, with higher numbers in the summer (F _1 32_ = 14.528, *P* < 0.001) (Fig 7). However, pupal abundance did not vary between seasons (F _1 32_ = 1.861, *P* = 0.172).

## Discussion

We previously demonstrated that gravid female *Culex* mosquitoes preferred to lay eggs in black dyed water [4]. However we show here that blue and shadow (colourless) pond dyes had no impact at all on oviposition in either laboratory tests or in the semi-field study in the tent. Given these results, it would seem that the black dye colour does have more attractive or stimulant properties than either the blue or shadow dyes. Possible explanations for these results are that mosquitoes choose to oviposit in black water because i) it indicates depth and therefore a lower threat of desiccation before juveniles develop, ii) it might indicate a higher concentration of organic matter providing nutrition [25, 26], iii) it mimics shading of the water body [27] and iv) black dyed water holds heat longer than undyed and mosquitoes may be able to visually sense near-infrared radiation (700 to >900 nm) [23].

Although there was no sustained effect on egg laying, blue and shadow dyes had an impact on *Culex* sp mosquito survival. For both dye treatments, significantly fewer adults emerged from containers that had been placed outside and covered to prevent colonisation by other mosquitoes or macroinvertebrates. These results are similar to those previously reported by Ortiz and Callaghan [4] (using an identical experimental design) where the number of adults emerging from a black dye treatment were significantly lower than the control. This was not easily explained since a toxicological assay found no significant larval mortality following exposure to any of the dyes, at various concentrations over a 48 hour period. If the experiment had been in a treated natural pond full of algae, and if that algal population was impacted by the treatment, then an explanation of the results might be a reduction in the availability of food, since algae form a significant proportion of the larval diet. However, mosquito larvae are not discriminatory and their diet will consist of detritus and microorganisms as well as algae [28]. In this particular experiment, tap water was used with guinea pig food (and, potentially, resulting microorganisms) for larvae to eat. Therefore algae should not have been a limiting factor.

Our previous work on the effect of black pond dye failed to detect any measureable impact on mosquito abundance when the experiment was conducted in a naturally colonised container rather than under controlled conditions [4], despite a very strongly significant impact on survival and oviposition under controlled conditions. However, in contrast, the shadow dye treatment had a significantly negative impact on pupal abundance in a naturally colonised container in late summer. The containers all had high abundances of *Culex pipiens* mosquitoes which generates competition and can have a significant impact on the development rate [29–31] and survival of conspecific mosquitoes [17, 19, 32]. It is likely that, since these dyes are not oviposition attractants, a lack of difference between treatments (apart from the impact on pupae during one season) is more related to overcrowding and competition having a greater impact than the dye. The relationship between mean larval numbers converted into mean pupal numbers shows a very low survival rate (between 1% minimum and 7% maximum) compared to the emergence rates in the controlled experiment (around 60%).

Vision is a long-range cue used for oviposition site location by many mosquito species [33]. A number of studies have looked at the behavioural ecology of oviposition choice, including colour, for a variety of mosquitoes of the genera *Aedes*, *Culex*, *Anopheles*, and *Toxorhynchites* [16, 18, 25, 34–37]. Black, blue and red colours all seem to be attractive to species including *Aedes albopictus*, *Culex annulirostris*, *Aedes albopictus* and *Aedes aegypti* [25, 35]. However many of these studies result from laboratory studies, which whilst instructive, may not accurately reflect the cues that are used in the field. Whilst laboratory studies that measure the total number of mosquito eggs (or egg batches) laid in test versus control conditions can provide useful information on oviposition stimulants and repellents, these studies can say little about the impact of these chemicals on oviposition in nature. This has certainly been the case in our experimental research.

It has been suggested that the terminology used in laboratory and field studies should be clarified, so that oviposition attractants or repellents are terms used when mosquitoes are using long- to middle-range cues resulting in a reorientation of flight direction [38]. In the case of short-range or contact cues, such as those used in laboratory studies such as ours, Day [35] suggests that the term stimulant or deterrent would be more accurate.

## Conclusion

These results show that pond dyes have an impact on mosquito behaviour and survival. Although the blue and shadow dyes had no impact on oviposition (unlike black dye [4]), the emergence of adults in dyed water was significantly impacted. These results do imply that the dye is in some way toxic to the mosquitoes over a long period of time, although it is not clear what is happening.

Populations of mosquitoes are likely to change as landscape and climate changes, and it has been suggested that towns and cities represent some of the highest risk areas for potential transmission of bird-related mosquito-borne diseases [39]. The ornithophagic habit of *Cx*. *pipiens* limits its potential as a bridge vector but seasonal abundance and other eco-behavioural characteristics predispose this species to serve as a potential enzootic vector of WNV, capable of maintaining cycles among bird populations, in the UK [40]. It is important to understand environmental factors that might impact on mosquito population success in urban habitats, particularly if these factors are anthropological in nature. The results presented here and in our previous work show that dyes are not totally neutral and can reduce fecundity as well as act as attractants [4]. Mosquito larvae are normally one member of a freshwater ecosystem that includes other macroinvertebrates. We know that these interact with each other and so the next stage will be to look at mosquito populations in dyed ponds containing whole communities.

## Supporting information

S1 Table Raw data for experiment to measure number of egg batches laid by *Culex* spp. in semi-field conditions and in light or dark conditions in the laboratory. (XLSX)

S2 Table Raw data for experiment to measure adult emergence from the three treatments (tap, blue and shadow) in summer and autumn. (XLSX)
